# Modelling and Optimization of the Processing of a Healthy Snack Bar Made of Grape and Tomato Pomaces

**DOI:** 10.3390/foods11172676

**Published:** 2022-09-02

**Authors:** Rocío Santiago-Ramos, Cristina L. M. Silva, Inês N. Ramos

**Affiliations:** CBQF—Centro de Biotecnologia e Química Fina—Laboratório Associado, Universidade Católica Portuguesa, Escola Superior de Biotecnologia, Rua Diogo Botelho 1327, 4169-005 Porto, Portugal

**Keywords:** byproducts, tomato pomace, grape pomace, circular economy, snack, convective air dryer, thin-layer modelling, focus group, texture, water activity

## Abstract

A snack made of 36% by byproducts of grape and tomato pomaces was developed, also including other ingredients, such as oats, chia, quinoa, honey and peanut butter. The recipe was defined as tasty and healthy by a focus group. The snack was produced by using forced air at three different drying temperatures (50 °C, 60 °C and 70 °C). The Newton, Page, Henderson and Pabis, and Midilli–Kucuk models fit the drying curves well. The average values for the Newton’s model drying constants were *k*_50_ = 2.71 × 10^−1^ ± 3 × 10^−3^ min^−1^, *k*_60_ = 2.76 10^−1^ ± 4 × 10^−3^ min^−1^ and *k*_70_ = 3.91 × 10^−1^ ± 8 × 10^−3^ min^−1^ at 50 °C, 60 °C and 70 °C, respectively. The product’s quality was assessed in terms of storage with respect to water activity and texture (hardness, springiness, cohesiveness, chewiness and resilience). There were no differences among the three tested processing temperatures in terms of their influence the final product’s quality. As there were no significant differences between initial and final water activity and texture attributes at any temperature and they were mainly unaltered during storage, the snack bar was considered stable during this period. This new snack, which includes byproducts from the food industry, reduces food waste and contributes to a circular economic model, simultaneously presenting environmental and economic advantages.

## 1. Introduction

On average, each person in Europe wastes 173 ± 37 kg of food per year, of which 11% comes from food processing, according to an estimate from 2012 [1]. Food waste not only impacts the unavailability of food but also generates energy and human resource waste, affects climate change, with a negative economic impact [2]. It is both a challenge and a responsibility for food scientists to reduce food waste. In 2015, food waste was considered a priority in the EU Circular Economy Action Plan [2]. The food waste generated during manufacturing and processing can be avoided by correctly handling and storing products, selecting appropriate packaging or increasing the shelf life of products [3]. An alternative way to prevent food waste is by transforming byproducts into actual food products. Byproducts are materials generated during a manufacturing process that are not the primary goal of the process. However, they are usually a good source of nutraceuticals and possess components that are beneficial for human health [4].

Most industries generate byproducts; for instance, the meat industry generates bones, fat and viscera; the dairy industry separates whey and curd; and the wine industry creates pomace residues after the trimming process. The world’s production of wine totalled 292 million hectolitres (mhL) in 2018, with Portugal in the 11th position in the ranking of major wine producers, accounting for 6.1 million hL in 2018 [5].

Grape pomace is estimated to account for 20% of the fruit’s total weight, and disposal of this residue is a challenge for the industry [6]. Traditionally, grape pomace was used to distil fortified wines and as fertilizer or animal feed. However, these uses present a negative side, as they can affect crop yields and animal weight gain [7]. New alternatives that fully exploit the potential of grape pomace, revalorizing it, are emerging. Some of these alternatives include the production of biosurfactants, pullulan (an exopolysaccharide produced by a fungus that can be used as a low-calorie bulking additive) or biosorbent material for the removal of heavy metals from water [8]. The grape pomace obtained as a by-product is rich in dietary fibre and is a source of protein. It is also a source of natural antioxidants and compounds that can be used in food, such as phenolic compounds, flavonoids and anthocyanin pigments [9].

Something similar occurs with tomato processing industries, which also generate pomace. More than 186 million metric tons (mT) of tomatoes were produced worldwide in the year 2020 [10]. Regarding the tomato processing industry, 38.78 million tons were processed in 2020, with 1.26 million tons processed in Portugal [11]. Tomatoes are generally processed into juice, soup, concentrates, sauces, puree, dry tomato, ketchup or tomato paste [12]. The generated byproducts represents 5% of the tomato weight [13] and is generally disposed of as solid waste, although they are sometimes used for animal feeding [14]. Tomato byproducts are mainly composed of peel, pulp and seeds and are considered to be high in dietary fibre (59.0% on a dry weight basis (d.b.)) and a source of protein (19.3% d.b.) [15]. Moreover, recent studies have shown that tomato pomace has considerable nutraceutical value, as it has a high content of carotenoids. The primary carotenoids identified in tomato peels are lycopene, phytoene, phytofluene, β-carotene, cis-lycopene and lutein [16]. Lycopene is an antioxidant that is more powerful than other carotenoids in reducing the risk of atherosclerosis and coronary heart disease and possibly preventing cancer [12]. Tomato lycopene is convenient as a natural food colorant, as it is active at low concentrations, stable when submitted to heat and extreme pH values in food processing, presents no off-flavours and goes through an entire colour spectrum from yellow to deep red [16]. Moreover, tomato seeds are rich in oil, presenting with between 10–12% and 17–18% (*w/w* of dry seed) oil content under mechanical extraction and 20–23% oil content (*w/w* of dry seed) by solvent (petroleum ether) extraction, with promising health benefits, such as prevention of hypercholesterolemia due to policosanol and plant sterol contents [17].

Because both tomato and grape pomaces are nutritious, using them to manufacture a product for human consumption is interesting. In a comprehensive review of the several uses of tomato pomace, the effects of its incorporation into various foods were summarized, namely crackers, cookies, bread, tarhana, snacks, pasta, hamburgers, sausages and tomato paste [18]. New trends in the reuse of pomace suggest its use in snack manufacturing [19]. Some recent studies have suggested different ways of creating snacks using grape or tomato pomace. Researchers combined tomato pomace with barley flour by blending them into a paste upon extrusion [20]. Another study showed promising results with a similar process, but the mix included corn and rice flour [21]. Regarding grape pomace, it can also be transformed into flour [22] and incorporated in the production of, e.g., cereal bars [23], cakes [24], pasta [25] and chocolate spread [26]. No relevant literature was found describing the use of grape and tomato pomace in the manufacture of a snack bar.

A review of trends in snacks reveals that the snack bar industry is a growing market worldwide that is projected to record a CAGR (compound annual growth rate) of 4.45% between 2020 and 2025. There is an increasing demand from consumers for on-the-go, healthy snack bars. Snack bar consumption data show that consumers tend to purchase snack bars that present functional claims [27]. In the production of snack bars, other ingredients should be considered. It is important to include one or more binder ingredients that act as “edible glue” ingredients, fortifying the bar so it does not crumble [28]. In the case of cereal bars, oats are the main ingredient associated by consumers [29]. However, the use of innovative, healthy ingredients is becoming increasingly common.

Regarding the manufacturing process in the industry, the most commonly used unit operations in processing fruit snack bars are drying with a structured gel matrix, sponges or scaffolds and extrusion. Drying is the most used and traditional process, consisting of applying heat to evaporate water from the product. Various types of drying can be applied, such as infrared, freeze drying, convective hot air drying or solar drying. Convective drying is the most commonly used method in industry for mass production [19]. When designing the production process of a product at the laboratory level, it is important to determine how to upscale it for the industry. Therefore, it is essential to understand the kinetics of the drying process, and theoretical and semi-theoretical models play an important role.

The main focus of this research was to develop a healthy and consumer-appealing snack bar made of byproducts of grape and tomato pomace produced in a convective air dryer. The impact of three processing temperatures on the physicochemical properties of the final product was assessed, and different thin-layer drying models were fitted to experimental water content values over time. Finally, the stability of the product was monitored under storage conditions for the tested processing temperatures.

## 2. Materials and Methods

A recipe was developed using the feedback provided by a focus group. Three replicates were produced for each of three processing temperatures. The colour, water content, water activity and texture of the final products were assessed. Additionally, water activity and texture were evaluated under storage conditions one, two, three and five weeks after production.

### 2.1. Focus Group

Two male and six female volunteers of varying nationalities, aged 20 to 30, and non-smokers, participated in a focus group. The goal was to identify the strengths and weaknesses of a snack bar prototype in terms of aspects such as flavour, appearance and how healthy and sustainable the product was perceived by the consumer [30]. The prototype was similar to the final recipe described in Section 2.2 and presented in Table 1, except that chia seeds (2.3% in mass) were not soaked in water. The following topics and subtopics were discussed in the three focus group sessions: (a) participant profile (consumption of snacks, concerns about health and concerns about sustainability), (b) sensory evaluation (appearance, smell, flavour and texture) and (c) final comments (product perception as healthy and detection of byproducts).

### 2.2. Preparation of the Samples

Grape pomace (white seedless Thompson cv.) and tomato pomace (Roma cv.) were used as the two core ingredients of the bars, comprising as much as 40% of the total weight of the mix. The pomaces were obtained in the laboratory by manual pressing with a potato masher, straining the liquid component through a 1.5 mm mesh sieve to a large container and waiting 30 min to strain completely. They were added to a mix of white quinoa seeds (Ignoramus Produtos Naturais Lda., Samora Correia, Portugal) and oat flocks (flocos de aveia finos, Áreaviva, Modelo Continente Hipermercados S.A., Matosinhos, Portugal), and manually stirred until homogeneity was achieved. Oat flocks and quinoa seeds were used as dry ingredients to compensate for the wetness of the pomaces. Three cohesive ingredients were added: honey (mel de flores, Modelo Continente Hipermercados S.A., Portugal), peanut butter (extra smooth Skippy creamy, Hormel Foods Corp., Austin, MN, USA) and chia seeds for the gum (Áreaviva, Modelo Continente Hipermercados S.A., Portugal). Chia seeds were soaked in water (125 g of water per 25 g of chia) for a period of two hours to exudate the maximum amount of gum [31]. The ingredients are detailed in Table 1.

### 2.3. Experimental Procedure

**Figure 1:**
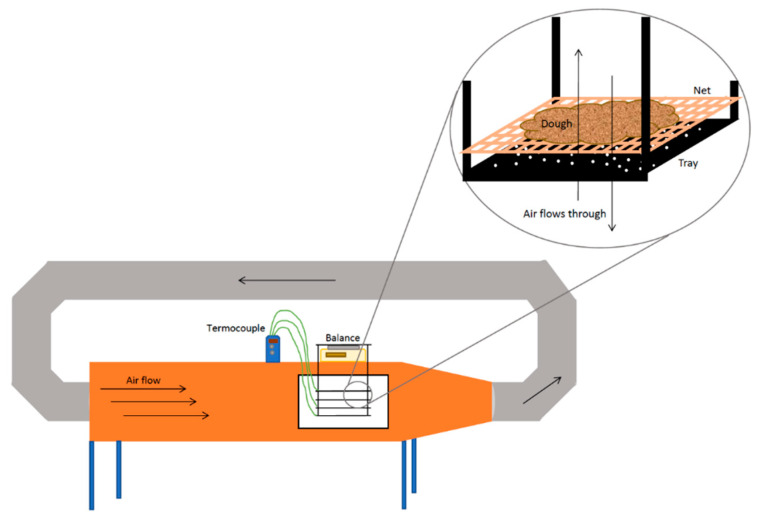
Convective tray-dryer setup.

A convective tray dryer (Armfield UOP8, Ring-wood, England) was used for processing, as depicted in Figure 1. 

The airflow was set to a constant speed of 0.54 m/s for all the experiments and was measured with a vane anemometer with ±0.01 m/s accuracy (LCA 6000, Airflow Instrumentation, Buckinghamshire, England). Three processing temperatures of 50 °C, 60 °C and 70 °C were chosen, and nine cycles were carried out, with three replicates for each temperature. The temperature inside the dryer was measured using a thermocouple (Datalogger Thermometer HH374, Omega, Norwalk, CT, USA) with momentary fluctuations of ±5 °C. An amount of 100 g of the mix was added to each of the three trays. The total mass was measured throughout the process and recorded automatically every 5 min with a precision balance (Sartorius BP6100, Goettingen, Germany) connected to a computer. 

Every cycle was finished once the estimated final water content (Xf) was around 11% on a wet basis, with this value established after several trials in which it corresponded to a_w_ ≤ 0.6, a value generally recognized as microbiologically safe [32]. The processed samples consisted of a sheet of approximately 20 × 30 cm and that was cut into 3 × 10 cm snack bars for posterior packaging.

### 2.4. Water Content and Water Activity

The water content of the snack bar samples was measured following method 984.25 from AOAC International [33]. The samples were dried in an air oven (Ehret, Emmendingen, Germany) at 104.5 °C until constant weight was achieved after approximately 7 h. A digital balance (Mettler Toledo AM100, Greifensee, Switzerland) with a sensitivity of ±0.0001 g was used. In order to obtain a single representative value, the outliers that provided a coefficient of variation (CV) ≥ 0.1 were removed. Water activity was determined immediately after processing and after one, two, three and five weeks of storage using a Hygrolab meter (AquaLab 3TE, Decagon Devices Inc., Washington, DC, USA). All measurements were carried out at room temperature (23.0 ± 1 °C) and performed in triplicate.

### 2.5. Packaging and Storage Conditions

A 0.04 mm foil that consisted of reversible metalized polypropylene was used to package the snack bars. Envelopes of approximately 5 × 12 cm were made with a sealer (FS 3261 Clatronic International Gmbh, Kempen, Germany). The packages were stored away from light in a dry environment at room temperature (23.0 ± 1 °C).

### 2.6. Computer Vision System (CVS) for Colour Evaluation

Pictures of the samples were taken in a HAVOX HPB-40D photo studio box measuring 43 × 43 × 43 cm. Two 30 W parallel, dimmable LED lights measuring 43.5 cm in length were placed at a 90° angle 40 cm above the base for illumination. The colour temperature was 5500K, with a colour rendering index of 93+ and a luminous flux of 13,000 lumens. The images were taken with a Canon colour digital camera (USA, model EOS 60D) with a Canon EF-S 18-55mm f/3.5-5.6 IS II lens. The selected ISO was 100, the focal length was 18 mm, the camera opening was 6.625, the exposure time was ½ s and the f value was 1/10. 

The pictures were processed, and values for the parameters L*, a* and b* were obtained using MATLAB R2018a software (MathWorks Inc., Natick, USA). Calibration curves were built for L*, a* and b* parameters and used to correlate the values obtained by processing the images with the software, as well as the experimental values obtained with a colourimeter (Minolta CR-400, Konica-Minolta, Osaka, Japan). To this end, pictures of 26 colour standards were taken on a black background inside the box with the same illumination. The TCD (total colour difference) was calculated between the samples before and after processing (Equation (1)), which corresponds to the distance in the colour space between two given colour points using the coordinates L*, a* and b*: colour 1 (L_1_*, a_1_* and b_1_*) and colour 2 (L_2_*, a_2_* and b_2_*) [34].

(1) $$\mathrm{TCD}=\sqrt{{\left(L_{2}^{*}-L_{1}^{*}\right)}^{2}+{\left(a_{2}^{*}-a_{1}^{*}\right)}^{2}+{\left(b_{2}^{*}-b_{1}^{*}\right)}^{2}}$$

### 2.7. Texture Profile Analysis

A TA.XT Plus texture analyser (Stable Micro Systems, Surrey, UK) with a 30 kg cell was used, with a cylindrical stainless-steel probe (P/50) with a flat end. In order to simulate the act of chewing, texture profile analysis (TPA) was performed by compressing and decompressing the snack bar twice and measuring the force. The applied force (N) corresponded to a deformation of 50% of the sample height. The configuration parameters were pre-test speed of 2 mm/s, test speed of 1 mm/s and post-test speed of 1 mm/s. The measurements were performed in triplicate for each bar. The software used for visualization of the texture profile curves was Texture Exponent 32 (Surrey, UK), which also allowed for determination of various texture attributes, such as hardness, springiness, cohesiveness, chewiness and resilience.

### 2.8. Statistical Analysis

Differences in TCD, a_w_ and texture parameters between the samples were statistically analysed through one-way analysis of variance (ANOVA) and non-parametric Kruskal–Wallis H test for non-normal data, Tukey and Mann–Whitney ad hoc tests. These tests were performed to separately analyse the effects of storage and temperature. A significance level of 5% was assumed for all analyses. IBM SPSS Statistics for Macintosh was used for data analysis (version 25.0. 2017, New York, NY, USA). 

### 2.9. Drying Modelling

To mathematically describe the processing of the bar, drying models were used, as the process mainly consists of water loss. Numerous thin-layer equations used to model drying curves have been compiled by other authors [35,36], and those used in the present study are expressed in Table 2.

X_i_ is the water content on a dry basis for any given time (I), and X_0_ is the initial water content. The equilibrium water content on a dry basis (X_e_) and drying parameters (k, k_1_, k_2_, a, b and N) were estimated for each model by non-linear regression analysis using the IBM SPSS Statistics for Macintosh (version 25.0. 2017, New York, USA). The 95% standard error of the parameters, coefficient of determination (R^2^) and root mean square error (RMSE) were also calculated. The statistical criteria for choosing the best model were the highest values of coefficient of determination (R^2^) and the lowest values of the standard deviation of the experimental error (s) [37]. The sum of RMSE for the various replicates and temperature conditions was determined, as well as the sum of R^2^.

## 3. Results

### 3.1. Focus Group

Understanding consumers’ perception of a product provides an indication on its potential success in the market. A focus group is a qualitative tool used not only to determine consumers’ perceptions of flavour but also to elucidate their overall opinions of a product [30]. Focus groups are frequently used to tackle a product’s weaknesses and improve it according to consumer preferences [38]. The opinions generated from the three focus group sessions are summarized in Table 3.

All the participants were consumers of snacks (either regularly or on occasion), and in general, they cared about health and sustainability. Five participants rated the bar nice and appealing to the eye. Three participants stated that the bar looked healthy. The smell was perceived as nice and pleasant by all participants. Five participants identified peanut notes. All participants described the flavour as well-balanced, and five participants also rated a perfect degree of sweetness in the flavour. They all agreed that the flavour was more grainy than fruity, but only two participants remarked that they would prefer more fruits in it. Three participants remarked on a bitter aftertaste coming from the peels of the fruits but perceived this aftertaste as positive. The texture of the bar was negatively evaluated in general because it was very brittle and crumbled when held in the participants’ hands. All the participants agreed that this was the weakest point of the product and that it should be improved. 

All participants concluded that the product was perceived as healthy in appearance and flavour. They remarked that it was not noticeable that the snack was made using byproducts and stated that it was a pleasant surprise for them. Because the appearance, smell and flavour were so highly rated by the participants, the ingredients and quantities used remained the same in the final formulation as in the first prototype. Only the texture was defined as a characteristic that should be improved. A cohesive ingredient was needed in the mix to improve the consistency. To this end, the gumming properties of chia were considered, and seeds were soaked in water as described in Materials and Methods. This was one of the key points that improved the consistency of the bar, affording a robust, non-crumbling snack. 

### 3.2. Estimated Nutritional Composition of the Product

The estimated nutritional composition of the snack bars is described in Table 4 per 100 g and per 20 g portion according to the original nutritional composition of the ingredients based on their weight. The original nutritional composition was described on the package labels for all the ingredients, except for grape and tomato, the information of which is presented on Nutrition Data Home Page [39], based on data from the USDA National Nutrient Database for Standard Reference. The RDI (recommended daily intake) was calculated according to FAO (Food and Agriculture Organization of the United Nations) recommendation for an adult 2000 kcal diet [40]. The nutritional features listed in Table 4 were not altered by the use of low processing temperatures (50 to 70 °C), as reported by Fellows [41]. The resulting product comprised 36.4% byproducts.

According to the FAO guidelines, the resulting bars satisfy some nutritional claims [42] and can be declared a source of protein (>5 g protein/100 g product) and dietary fibre (>3 g fibre/100 g product), with low contents of saturated fats (<1.5 g saturated fats/100 g product). 

### 3.3. Drying Kinetics Modelling

**Figure 2:**
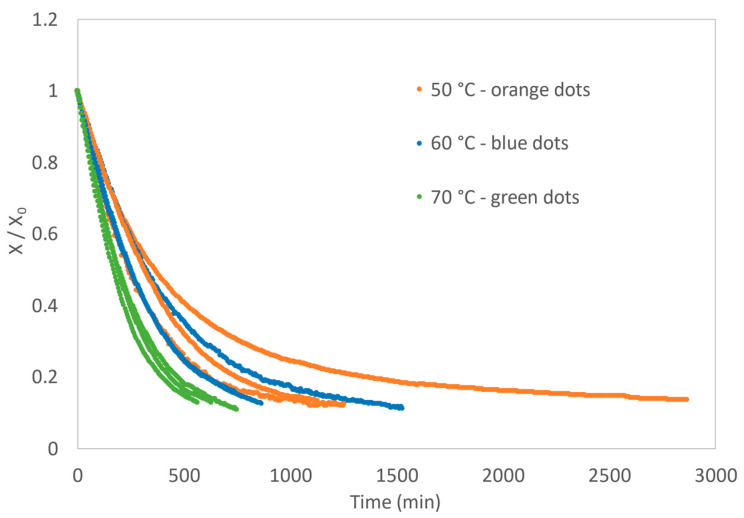
Normalised experimental data of the three replicates for the three tested drying temperatures.

The initial water content of the samples (X_0_) was 47.1% ± 1.4 on a wet basis. Figure 2 shows the normalised water content for the nine experiments; as expected, the higher the temperature, the shorter the average processing time. The average processing time was 29.2 min at 50 °C, 17.4 min at 60 °C and 10.8 min at 70 °C.

The models expressed in Table 2 were fitted to the experimental drying curves obtained by processing the samples at the three drying temperatures. The values of the coefficient of determination (R^2^) and standard deviation of the experimental error (s) were used as statistical criteria. The two-term and two-term exponential models did not present with normality and homoscedasticity of residuals and were therefore discarded. The fits of the Newton, Page, Henderson and Pabis, and Midilli–Kucuk models were all satisfactory, with coefficients of determination of R^2^ > 0.97 for all nine replicates. The Midilli–Kucuk model presented with slightly better fits, as it showed the highest values for the coefficient of determination (R^2^) and the lowest values for the standard deviation of the experimental error (s) for most replicates. However, for comparison of drying constants at the three tested temperatures, the Newton model was chosen for its mathematical meaning, as it does not include an exponential parameter (N) associated with the time variable.

**Figure 3:**
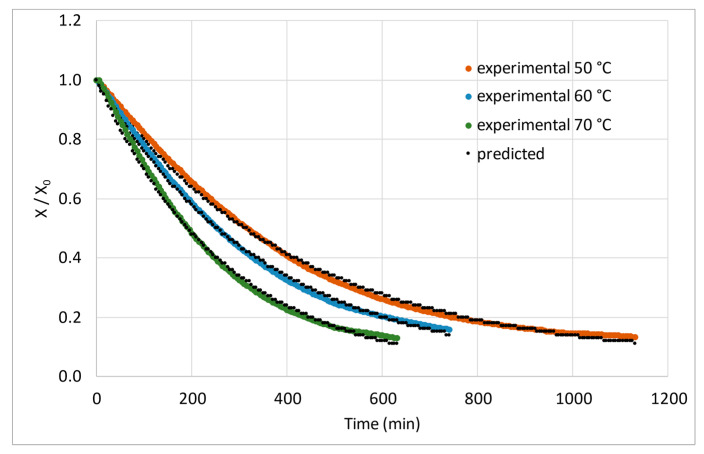
Normalised experimental data and values predicted by the Newton model for one replicate at each drying temperature.

Table 5 displays the drying constants (k in min^−1^) estimated with the Newton model, their 95% standard error of the parameter (SE) and the equilibrium water content on a dry basis (Xe in kg_water_ kg_dry matter_^−1^) for the three tested temperatures and each replicate. This table also presents the corresponding statistical indicators, *R^2^* and *s* values. An example of the plots of the Newton model for one replicate of the three drying temperatures is depicted in Figure 3, with normalised water content.

The following average values for the drying constants were obtained with the Newton model: *k*_50_ = 2.71 × 10^−1^ ± 3 × 10^−3^ min^−1^, *k*_60_ = 2. 76 × 10^−1^ ± 4 × 10^−3^ min^−1^ and *k*_70_ = 3.91 × 10^−1^ ± 8 × 10^−3^ min^−1^ at 50 °C, 60 °C and 70 °C, respectively, indicating that, as expected, the drying constants increased with increasing drying temperatures, corresponding to faster processing of the snack.

To the best of our knowledge, no studies have been reported in the literature involving the use of the Newton model to describe the drying kinetics of snacks, although some studies have involved its use it for pomaces or other foods. Kumar et al. [43] calculated a drying constant value of 1.524 × 10^−2^ min^−1^ by adjusting the Newton model to thin-layer drying of carrot pomace at 60 °C. Rayaguru et al. [44] obtained values of 4 to 5 × 10^−3^ min^−1^ for drying constants of thin-layer drying of stone apple slices at temperatures ranging from 50 to 70 °C. Bala et al. [45] obtained values of 2.9 to 8.27 × 10^−3^ min^−1^ for drying constants of tray drying of asparagus roots at temperature ranging from 60 to 70 °C. These authors presented drying constants for the Newton model of one or two orders of magnitude lower, possibly due to the products under study or the use of different air velocities, which was not always specified in the literature. Regarding diffusional models for apple pomace, effective diffusivity ranged between 5 and 91 × 10^−10^ m^2^s^−1^ for temperatures of 40 to 120 °C [46].

### 3.4. Colour Evaluation

Colour is a determining factor in a consumer’s choice of a product, sometimes playing a more important role than flavour itself. Understanding how the colour of a product affects a consumer’s decision is fundamental in marketing [47]. The average colour values of L*, a* and b* obtained by a computer vision system (CVS) before processing are presented in Table 6, together with their standard deviations and coefficients of variance (CVs). Because the total average of the CV is equal to 0.0813, values lower than 0.10 indicate that the samples did not differ significantly before processing, taking into account their irregular nature.

**Figure 4:**
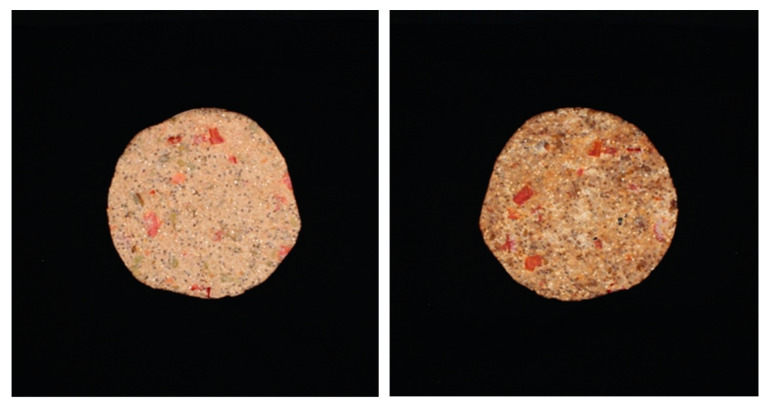
Pictures of sample before (**left**) and after processing (**right**).

After processing, the mean of the total colour difference (TCD) values was 10.46 ± 1.86, meaning, according to Dr. Lange’s colour classification [48], that the variations before and after processing represent a considerable visual difference. An example of this difference in which browning is also notorious is illustrated in Figure 4.

Colour can also change in a specific direction, meaning that one of the parameters (L*, a* or b*) changes more than the others. The total average change before and after processing for L* was $\bar{\left(L_{2}^{*}-L_{2}^{*}\right)\;}$ = −9.80; for a*, it was $\bar{\left(a_{2}^{*}-a_{1}^{*}\right)\;}$ = 2.77, and for b*, it was $\bar{\left(b_{2}^{*}-b_{1}^{*}\right)\;}$ = 2.48. The change in L* had a negative value, meaning that the bar sample became darker as a result of processing. The average difference for a* indicates that the bar tended to present with green tones, and the average difference in b* indicates that it tended to present with blue tones. Regarding temperature groups, TCD no statistically significant differences were observed (*p* > 0.05), so the processing temperature did not impact colour change. 

### 3.5. Water Activity Evaluation

**Figure 5:**
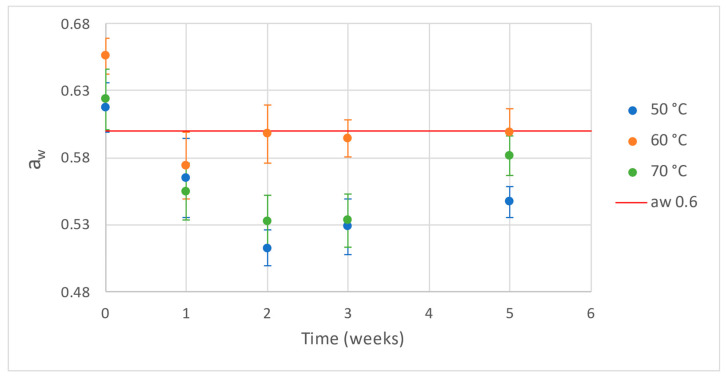
Average water activity values and confidence intervals for the three drying temperatures. The red line represents a_w_ = 0.6.

Water activity (a_w_) is a parameter commonly used in the food industry as an indicator of the possible growth of various microbes. A value of a_w_ ≤ 0.6 is generally recognised as safe from a microbiological point of view [32]. When packaging the bars, the initial water activity content was close to 0.6, which was the target value. For all the processing temperatures, the a_w_ value dropped one and two weeks after packaging, with a slight increase from weeks three to five for bars processed at 70 °C (Figure 5).

In order to statistically compare the three different temperature groups, a_w_ data obtained for each week and replicate were normalised according to their initial values. Significant differences were observed for the three temperature groups, although only at weeks two and three. However, independent of processing temperature, water activity tended toward equilibrium, with a similar common value for all temperature groups from week three onwards. For the samples processed at 50 °C, significant differences in a_w_ values were recorded between weeks zero and two and between weeks zero and three. The same differences were observed for samples processed at 70 °C. For the samples processed at 60 °C, significant differences were recorded in a_w_ values between weeks zero and one. Because no significant differences were recorded in a_w_ values between weeks zero and five for any temperature, there seems to be some stability in the product. However, the changes in a_w_ occurred indicate that at the beginning of storage, the bar loses water to the environment, which can be interpreted as the bar tending toward a water equilibrium inside the packaging.

### 3.6. Texture Profile Analysis (TPA)

The texture of a product is an indication of its quality. Previous studies showed that the results obtained from TPA analysis correlate well with the results of sensory analysis panels [49]. In this study, the parameters of hardness, springiness, cohesiveness, chewiness and resilience were determined. Other studies on TPA of snack products presented results of springiness, gumminess, fracturability, hardness, cohesiveness and chewiness of extruded oat–corn puffs [50], as well as those of brittleness, breaking strength and hardness of extruded maize–soybean puffs [51].

**Figure 6:**
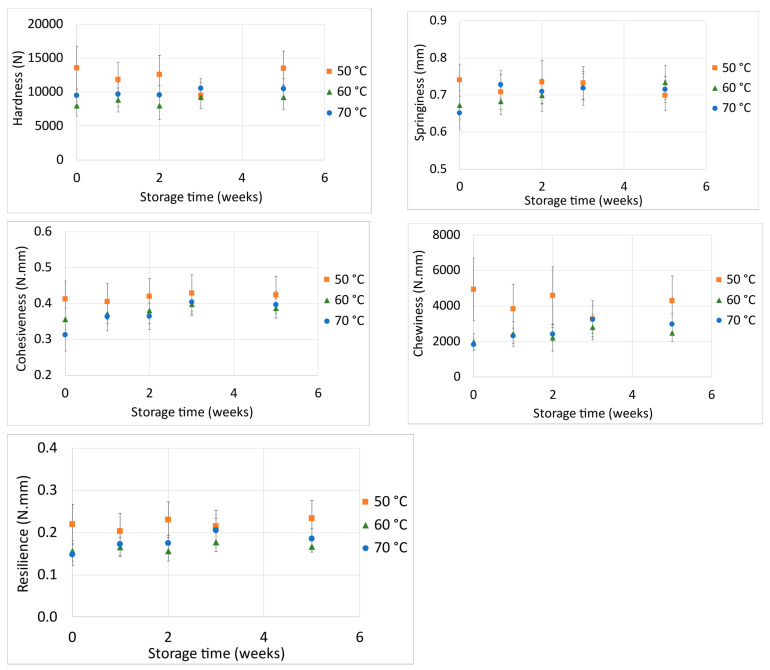
Hardness (N), springiness (mm), cohesiveness (N.mm), chewiness (N.mm) and resilience (N.mm) of bars processed at different temperatures relative to storage time (weeks).

In order to statistically compare the texture attributes during storage for each drying temperature, all data for each week and replicate were normalised according to their initial values. To assess whether there were specific significant differences between the different temperatures, the obtained measured data were compared week by week. The behaviours of the snack bar’s hardness, springiness, cohesiveness, chewiness and resilience during storage for each temperature are displayed in Figure 6.

Overall, all the attributes did not present with any significant difference (*p* > 0.05) during storage, independent of production temperature. This can be interpreted as a good indication of the product’s stability, also implying an appropriate choice of packaging material. Regarding the three processing temperatures used, there were few significant differences between the snack bar samples, with no clearly observable tendency for any of the texture parameters. These few significant differences are described blow. At the beginning of storage (week zero), the samples processed at 50 °C differed significantly from those processed at 70 °C, presenting with higher hardness, springiness, cohesiveness and chewiness values. Furthermore, at week zero, the samples produced at 50 °C were also differed significantly from those produced at 60 °C with respect to hardness and chewiness, presenting with higher values. At week two, the samples processed at 50 °C were differed significantly from those processed at 60 °C, presenting with higher hardness, chewiness and resilience values. Also at week two, the samples produced at 60 °C differed from those produced at 70 °C, with lower chewiness values. 

To the best of our knowledge, no texture studies on pomaces addressing processing temperature are available in the literature. Previous research on pomaces mostly assessed the effect of varying compositions on the final product. One possible interpretation is that once a lower processing temperature leads to higher total processing times, some mechanism contributes the snack’s hardness, springiness, cohesiveness and chewiness increase at week zero (keeping in mind that a similar final water content maintained for all the snacks). This is the opposite of what is expected due to case hardening at higher drying temperatures, whereby a hard outer layer is formed in the product. In this case, hardness would be higher at higher temperatures [52].

## 4. Conclusions

The sensory evaluation performed during the focus group sessions contributed to improving the product’s texture. Overall, the product was perceived as tasty and healthy. The byproducts, which constituted 36.4% of the total weight of the product, were not noticed as byproducts by the panellists. According to FAO guidelines, the resulting snack bar is a source of protein and dietary fibre, and it is low in saturated fats, according to its estimated composition, therefore presenting with many health benefits. The Newton, Page, Henderson and Pabis, and Midilli–Kucuk models presented with very good fits. The following average values for the drying constants were obtained with the Newton model: *k*_50_ = 2.71 × 10^−1^ ± 3 × 10^−3^ min^−1^, *k*_60_ = 2. 76 × 10^−1^ ± 4 × 10^−3^ min^−1^ and *k*_70_ = 3.91 × 10^−1^ ± 8 × 10^−3^ min^−1^ for 50 °C, 60 °C and 70 °C, respectively. 

Regarding colour, there was a considerable visual difference in the samples before and after processing. However, no significant difference was observed between the samples processed at the various tested temperatures; therefore, processing temperature did not affect the product’s colour. Water activity tended toward a similar value for all temperatures at week five, regardless of the production temperature used. This may be an indication of how the water activity tends toward an equilibrium under storage conditions. However, a longer storage time is suggested for further experiments to confirm this hypothesis. 

All evaluated texture attributes (hardness, springiness, cohesiveness, resilience and chewiness) were mostly stable under storage conditions. The preservation of initial texture and water activity possibly indicates that the chosen packaging material was appropriate for this product, as satisfactory stability was achieved over the first five weeks of storage. Moreover, no meaningful differences in the quality of the product were observed between the tested processing temperatures. 

Further shelf-life studies, including microbiological analysis of the product, are recommended to generate additional information on the product’s safety and quality under storage conditions. This proposed new snack, which includes byproducts from the food industry, reduces food waste and consequently contributes to a circular economic model, simultaneously presenting with environmental and economic advantages.

## Figures and Tables

**Table 1:** List of the ingredients used in the final recipe (for three batches).

Ingredient	Weight (g)	Weight (%)
Oat flocks	300	27.27
Dried quinoa seeds	50	4.55
Dried chia seeds	25	2.27
Honey	100	9.09
Peanut butter	100	9.09
Grape pomace	200	18.18
Tomato pomace	200	18.18
Water	125	11.36

**Table 2:** Thin-layer drying models fitted to drying curves.

Model	General Expression
Newton	$\frac{X_{i}-X_{e}}{X_{0}-X_{e}}=\exp\left(-k\cdot t\right)$ (2)
Page	$\frac{X_{i}-X_{e}}{X_{0}-X_{e}}=\exp\left(-k\cdot t^{N}\right)$ (3)
Henderson and Pabis	$\frac{X_{i}-X_{e}}{X_{0}-X_{e}}=a\cdot \exp\left(-k\cdot t\right)$ (4)
Midilli–Kucuk	$\frac{X_{i}-X_{e}}{X_{0}-X_{e}}=a\cdot \exp\left(-k\cdot t^{N}\right)+b\cdot t$ (5)
Two-term	$\frac{X_{i}-X_{e}}{X_{0}-X_{e}}=a\cdot \exp\left(-k_{1}\cdot t\right)+b\cdot \exp\left(-k_{2}\cdot t\right)$ (6)
Two-term exponential	$\frac{X_{i}-X_{e}}{X_{0}-X_{e}}=a\cdot \exp\left(-k\cdot t\right)+\left(1-a\right)\cdot \exp\left(-\;k\cdot a\cdot t\right)$ (7)

**Table 3:** Overview of the opinions of the participants in the focus group.

	Count	Panellist
**(a) Participant profile**		
Consumes snacks		
- Yes	4	1, 2, 3, 7
- No	0	
- Sometimes	4	4, 5, 6, 8
Cares about health		
- I would change my preference of a bar based on how healthy it is.	4	1, 3, 4, 8
Cares about sustainability		
- I would consume a product made from byproducts.	8	1–8
**(b) Sensory evaluation**		
Appearance		
- Nice, appealing	5	2, 4, 5, 6, 8
Smell		
- Peanut	5	2, 3, 5, 6, 8
Flavour		
- Right amount of sweetness	5	3, 4, 5, 7, 8
- Balanced, pleasant	8	1–8
Texture		
- Soft, crumbles	8	1–8
**(c) Final comments**		
Perceives the product as healthy		
- Yes	8	1–8
- No	0	
Noticed it was made with byproducts		
- Yes	0	
- No	8	1–8

**Table 4:** Estimated nutritional composition of the product per 100 g and per portion and the RDI per portion.

	Nutritional Composition (100 g)	Per Portion (20 g)	% RDI Per Portion (20 g)
Energy (kcal)	246.5	49.3	2.5
Fats	8.3	1.7	2.4
Saturated fats	1.4	0.3	1.4
Carbohydrates	35.6	7.1	2.3
Sugars	15.3	3.1	3.4
Fiber	5.3	1.1	3.6
Protein	7.1	1.4	2.8
Salt	0.1	0.0	0.9
Fe (mg)	0.6	0.1	0.8
Zn (mg)	0.3	0.1	0.5
P (mg)	28.1	5.6	0.8
Mg (mg)	13.8	2.8	0.9
Ca (mg)	21.9	4.4	0.4
K (mg)	91.6	18.3	0.5

**Table 5:** Parameters of the Newton model for the replicates processed at 50 °C, 60 °C and 70 °C.

Temperature	Parameter	Replicate 1			Replicate 2			Replicate 3		
		Value	s	R^2^	Value	s	R^2^	Value	s	R^2^
50 °C	k (min^−1^)	2.36 × 10^−1^ ± 1.5 × 10^−^^3^	9.96 × 10^−3^	0.997	3.36 × 10^−1^ ± 3.5 × 10^−3^	1.06 × 10^−2^	0.998	2.41 × 10^−1^ ± 3.9 × 10^−3^	1.28 × 10^−2^	0.997
	Xe	1.47 × 10^−1^ ± 1.2 × 10^−3^			9.92 × 10^−2^ ± 2.9 × 10^−3^			4.96 × 10^−2^ ± 5.9 × 10^−3^		
60 °C	k (min^−1^)	2.44 × 10^−1^ ± 1.7 × 10^−3^	7.40 × 10^−3^	0.999	2.82 × 10^−1^ ± 6.0 × 10^−3^	1.12 × 10^−2^	0.998	3.01 × 10^−1^ ± 3.2 × 10^−3^	7.08 × 10^−3^	0.999
	Xe	8.43 × 10^−2^ ± 1.9 × 10^−3^			1.89 × 10^−2^ ± 9.4 × 10^−3^			4.07 × 10^−2^ ± 3.7 × 10^−3^		
70 °C	k (min^−1^)	3.78 × 10^−1^ ± 4.4 × 10^−3^	5.49 × 10^−3^	0.999	3.69 × 10^−1^ ± 1.0 × 10^−2^	1.47 × 10^−2^	0.997	4.28 × 10^−1^ ± 8.8 × 10^−3^	1.08 × 10^−2^	0.998
	Xe	4.35 × 10^−2^ ± 4.1 × 10^−3^			1.10 × 10^−2^ ± 1.1 × 10^−2^			2.53 × 10^−2^ ± 8.3 × 10^−3^		

**Table 6:** Average values for L*, a* and b* obtained before processing, with standard deviation and coefficient of variance (CV).

	Average Values	CV
L*	74.82 ± 1.79	0.0239
a*	5.56 ± 0.77	0.1383
b*	12.20 ± 1.00	0.0817
**Total average**	N.A.	0.0813

## Data Availability

The data presented in this study are available upon request from the corresponding author.
